# Bacterial-Assisted Extraction of Bioactive Compounds from Cauliflower

**DOI:** 10.3390/plants11060816

**Published:** 2022-03-18

**Authors:** Enrico Doria, Daniela Buonocore, Antonio Marra, Valeria Bontà, Andrea Gazzola, Maurizia Dossena, Manuela Verri, Cinzia Calvio

**Affiliations:** 1 Department of Biology and Biotechnology L. Spallanzani, University of Pavia, 27100 Pavia, Italy; daniela.buonocore@unipv.it (D.B.); antonio.marra03@universitadipavia.it (A.M.); valeria.bonta01@universitadipavia.it (V.B.); maurizia.dossena@unipv.it (M.D.); manuela.verri@unipv.it (M.V.); cinzia.calvio@unipv.it (C.C.); 2 Department of Earth and Environmental Sciences, University of Pavia, 27100 Pavia, Italy; andrea.gazzola@unipv.it

**Keywords:** *Bacillus subtilis*, cellulases, xylanases, bioactives, extraction, nutraceuticals, phytochemicals, sustainability, vegetable by-products

## Abstract

The market for nutraceutical molecules is growing at an impressive pace in all Western countries. A convenient source of bioactive compounds is found in vegetable waste products, and their re-use for the recovery of healthy biomolecules would increase the sustainability of the food production system. However, safe, cheap, and sustainable technologies should be applied for the recovery of these beneficial molecules, avoiding the use of toxic organic solvents or expensive equipment. The soil bacterium *Bacillus subtilis* is naturally endowed with several enzymes targeting complex vegetable polymers. In this work, a raw bacterial culture supernatant was used to assist in the extraction of bioactives using isothermal pressurization cycles. Besides a wild-type *Bacillus subtilis* strain, a new strain showing increased secretion of cellulases and xylanases, pivotal enzymes for the digestion of the plant cell wall, was also used. Results indicate that the recovery of compounds correlates with the amount of cellulolytic enzymes applied, demonstrating that the pretreatment with non-purified culture broth effectively promotes the release of bioactives from the vegetable matrix. Therefore, this approach is a valid and sustainable procedure for the recovery of bioactive compounds from food waste.

## 1. Introduction

The agri-food sector generates massive amount of waste that not only causes the loss of market profit but, more importantly, represents a waste of precious environmental resources, such as land, water, fertilizers, chemicals, and energy expended in the production phase [1]. Moreover, organic waste might pose potentially severe pollution problems as it decomposes in landfills, releasing nitrogen and phosphorus in aquatic and terrestrial ecosystems and emitting harmful greenhouse gases [2].

In line with the circular economy concept, fruit and vegetable waste might be turned into a valuable resource as a natural and unlimited supply of biologically active compounds, including vitamins, polyphenols, dietary fibers, glucosinolates, essential oils, and organic acids, among the others, with an enormous economic potential as nutraceutical, pharmaceutical, cosmetic, and agro-pharmaceutical ingredients [3,4]. However, to maintain the green connotation, the recovery of natural bioactive compounds from discarded agro-food materials must rely on methods which (i) do not generate more polluting waste than the disposal of the raw biomass itself, (ii) are safe for the final product, and (iii) guarantee high revenues for the entire value chain [5].

Among the new emerging techniques, Enzyme Assisted Extraction (EAE) of biomolecules represents one of the most environmentally friendly and safe methodologies which can be used either as a stand-alone technique, or as a pretreatment that increases the efficiency of a coupled extraction system [6,7,8]. EAE is based on the fact that enzymes help to weaken or deconstruct the plant cell wall in which most bioactives are entrapped, making encased compounds more accessible for extraction. The most used enzymes for this purpose are hydrolytic enzymes such as cellulases, (endoglucanases, cellobiohydrolases, β-glucosidases), hemicellulases (endoxylanases and β-xylosidases), and pectinases (polygalacturonases, and pectinesterases). Other hydrolysing enzymes (proteases, amylases, pullulanases, pectate lyases, etc.) might further support the release of precious and low-abundant secondary metabolites from cellular components [6,7,8]. However, the use of expensive commercial enzymes would take a prohibitive toll on the entire process, mining the economic viability of the extraction [6,7].

One of the most formidable enzyme-producers is the Gram-positive soil bacterium *Bacillus subtilis. B. subtilis* is already heavily exploited for the industrial production of several biocommodities, such as degradative enzymes, heterologous proteins, bio insecticides, and antibiotics, and its products are considered as Generally Recognized as Safe (GRAS) [9,10,11]. Its reputation as an industrial pillar is due to its simple nutritional requirements, its excellent fermentation properties over a wide range of conditions, its genetic plasticity, enabling the optimization of its biotechnological performances, and its efficient secretory system, allowing for the recovery of massive amounts of bio-products directly from the growth medium [9,10,11]. 

*B. subtilis* lives predominantly in the soil, and this type of habitat has evolutionarily shaped its genome, leading to the accumulation of a large array of genes associated with the ability to degrade complex carbohydrates from decaying vegetable biomass. According to the Carbohydrate-Active Enzymes database [12], the bacterium is endowed with several genes encoding secreted enzymes involved in complex carbohydrates and lignocellulose degradation [13]. Moreover, it also encodes several proteases and many other hydrolytic enzymes [11,14]. Thanks to the above-mentioned characteristics, *B. subtilis* spent growth medium represents an inexpensive, rich, and wide-range collection of GRAS enzymatic activities, ideally suited to break down the plant matrix and improve the release of bioactives.

The aim of this work was to evaluate the efficacy of a method for the sustainable extraction of bioactive compounds from vegetable waste based on a low-cost EAE pretreatment. The economic viability of the procedure relied on the use of crude *B. subtilis* culture supernatants, obtained from a wild-type (WT) strain and from a new *B. subtilis* strain, overproducing cellulases and xylanases (OS58). The sustainability of the process was maintained by carrying out the extraction process in an aqueous environment, avoiding the use of any polluting solvent. The substrate, cauliflower (*Brassica oleracea* L*. conv. botrytis* (L.) *Alef. var. botrytis* L.), rich in phytochemicals endowed with a large range of beneficial biological activities, derived from the quota of raw products that did not meet the qualitative standards for commercialization and were discarded by an industrial agro-food processing plant. 

## 2. Results

### 2.1. Bacillus subtilis Enzymes and Pretreatment

**Figure 1:**
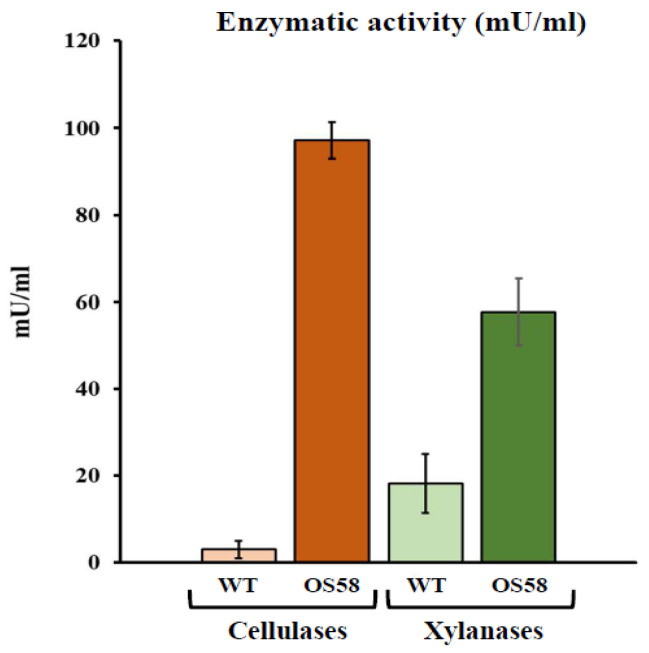
The enzymatic activity of cellulases (in orange) and xylanases (in green) in the WT and OS58 strains (as indicated on the x-axis). Cells were grown in a chemically defined medium for 24 h and assayed with a commercial kit. Enzymatic activity (in mU/mL of spent medium) is reported on the y-axis. Values represent the average of at least five independent experiments. Error bars represent the standard error of the mean (SEM).

To evaluate the effectiveness of the low-cost enzymatic pretreatment, two *B. subtilis* strains were used. The WT strain corresponded to the commonly used lab strain JH642 [15] in which the tryptophan and phenylalanine auxotrophies were cured and prototrophy restored. The second strain, namely OS58, was obtained by improving the intrinsic cellulolytic and hemicellulolytic propensity of *B. subtilis* through genetic engineering of the WT strain. The cellulases and xylanases activities released in the growth medium by the two strains were determined after 24 h incubation. The optimization process led to a drastic enhancement in enzyme secretion. As shown in Figure 1, the enzymatic units found in OS58 culture broth increased by over 30-fold for cellulases and 3-fold for xylanases compared to those released by the WT strain. These enzymes are supposedly playing a pivotal role in vegetable matrix breakdown, although a plethora of additional enzymes, which *B. subtilis* is known to secrete, might contribute to the final effect on the substrate [16].

### 2.2. Recovery of Phenolic Compounds 

In order to verify whether the crude enzymatic mixture released by the bacterial strains represented an effective pretreatment for improving the extraction of bioactive compounds, cauliflowers discarded from the food supply chain were collected from an industrial processing plant during different seasonal periods. The biomass, originated from different regions across Italy, was treated according to a standardized protocol. The processing began with the grinding of thawed cauliflowers into 0.2–0.5 cm^3^ pieces. The fragmented material was incubated at 50 °C with culture supernatants from either the WT strain, OS58, or the sterile growth medium, which was used as a negative control for the treatment. Subsequently, each mash was subjected to 60 cycles of pressurization at room temperature (20°–23 °C) for the extraction of bioactive compounds. Part of the liquid flow, collected from the apparatus after each extraction, was dried and resuspended in 50% methanol for the analyses of the nutraceutical compounds.

Important differences in the content of bioactives were observed among the four replicates, which were performed on different stocks of cauliflowers; such differences are presumably linked to seasonal effects and the geographical origins of the vegetable material. For this reason, the correlation among replicates was estimated by including the variable “replicate” as a random effect in the statistical model (see Materials and Methods).

**Figure 2:**
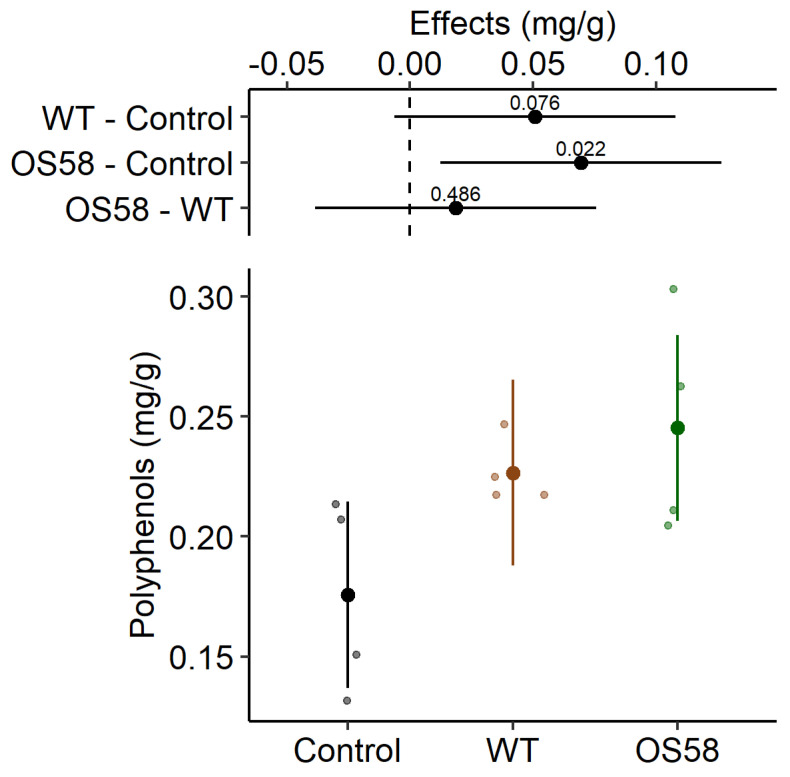
The total polyphenols content (calculated as mg per gram of fresh cauliflowers, before freezing) is reported for Control, WT, and OS58 groups as estimated means and 95% confidence intervals (larger dots and colored lines, respectively) by the linear mixed model (LMM). On the upper side of the plot, treatment effects are reported in mg/g as the estimated means and 95% confidence intervals for each comparison, as difference from the control or between enzymatic treatments, as indicated on the left (degrees of freedom-df = 10.7). The level of significance (*p*-value) is reported above each horizontal line; the dashed line corresponds to the “no effect” hypothesis. The values reported were obtained from the model using the emmeans package (n = 4, for each group) [17].

The total polyphenols recovered under the three conditions demonstrated that the enzymatic treatment improved the extraction efficiency (Figure 2 and Table 1). In particular, the polyphenols recovered upon treatment with the WT strain increased by 1.3-fold with respect to the control (*p* < 0.1, weak significance); however, the recovery further increased (by 1.4-fold with respect to the control, *p* < 0.05) when the treatment was performed with the optimized strain OS58, which is a better enzyme producer (Figure 2). The progressive increase in the recovery of polyphenols, observed by comparing the control, the WT, and the OS58 treatment, respectively (Table 1), suggests that the beneficial effect of the bacterial supernatants might be linked to the progressively higher amount of enzymes produced by the two strains.

#### 2.2.1. Catechins

**Figure 3:**
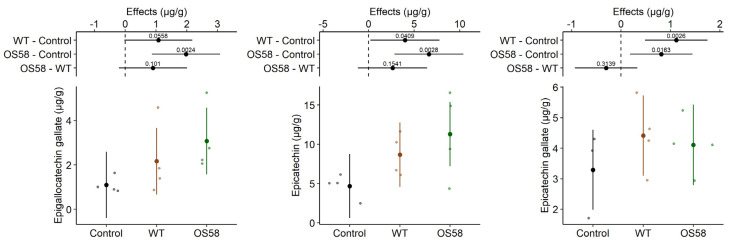
The catechins content (calculated as μg per gram of fresh cauliflowers) is reported for the Control, WT, and OS58 groups. A detailed description of these types of plots is provided in Figure 2.

The analysis of polyphenols was further deepened for the group of catechins. The HPLC profile for these compounds was evaluated in the treatment and control groups, revealing a significant improvement in their recovery, even with the WT strain (Figure 3). With respect to the control, the WT enzymes allowed the extraction of 2-fold more epigallocatechin gallate, 1.8-fold more epicatechin, and 1.3-fold more epicatechin gallate (Table 1). As observed for polyphenols in general, in all but the last case, the effect of the bacterial pretreatment appeared stronger with the optimized OS58 strain, where the yield with respect to the control raised by 2.8-fold for epigallocatechin gallate and by 2.4-fold for epicatechin, reinforcing the hypothesized link between the effect on the extraction efficiency and the amount of secreted enzymes. Conversely, the recovery of epicatechin gallate upon OS58 treatment (1.2-fold increase) was slightly lower than that obtained with the WT strain (1.3-fold higher than the control).

#### 2.2.2. Chlorogenic Acid

**Figure 4:**
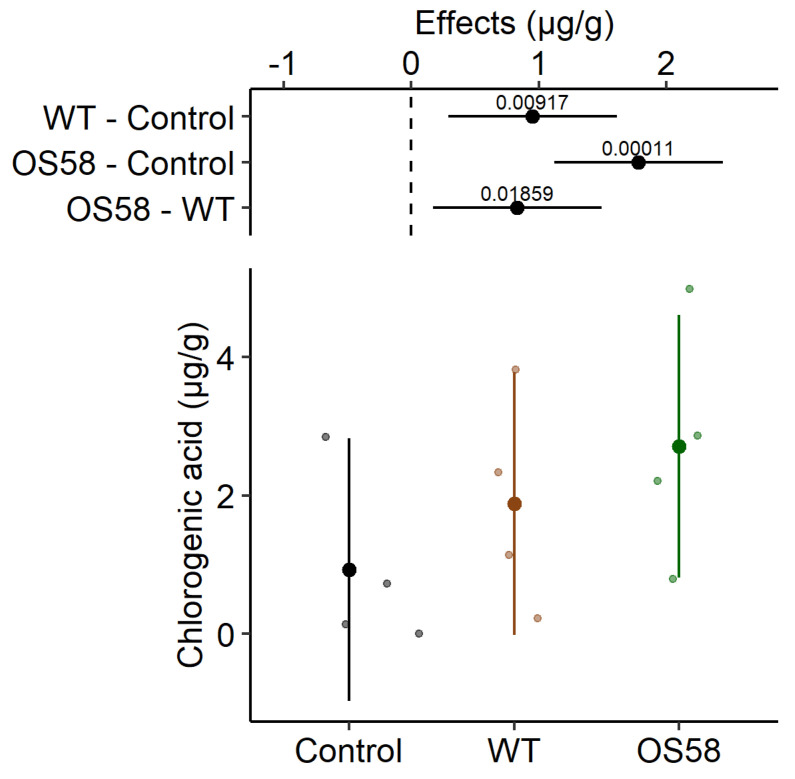
The chlorogenic acid content (calculated as μg per gram of fresh cauliflowers) is reported for the Control, WT, and OS58 groups. A detailed description of these types of plots is provided in Figure 2.

For the phenolic acids class of polyphenols, the recovery of chlorogenic acid was examined in detail in the three different extracts. As shown in Figure 4 and Table 1, the enzymatic treatment appeared to be extremely efficient in releasing the compound from the matrix. The enzymes produced by the WT strain were able to release 2-fold more chlorogenic acid than the control treatment, while following the treatment with the OS58 enzymatic pool, the recovery of chlorogenic acid was 2.9-fold higher with respect to the untreated control, validating the effectiveness of the enzymatic treatment. It is worth noticing that, for this compound, the difference between the OS58 strain and the WT achieved a 95% statistical significance (*p* = 0.019), corroborating the hypothesis that the effect is indeed due to higher amount of enzymes released by the optimized strain.

### 2.3. Recovery of Sulphur-Containing Plant Secondary Metabolites

#### Isothiocyanates

**Figure 5:**
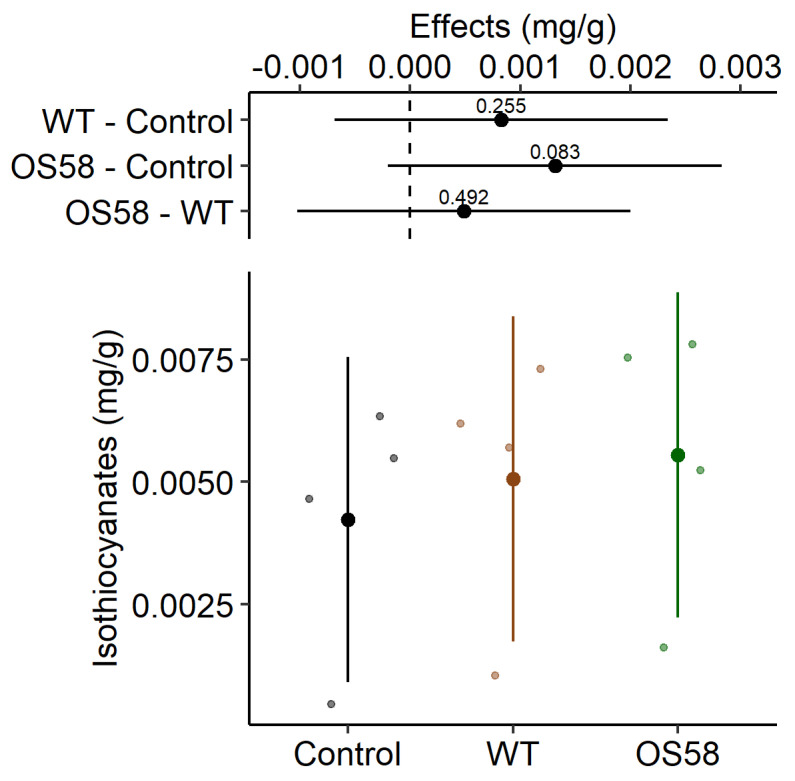
The isothiocyanates content (calculated as mg per gram of fresh cauliflowers) is reported for the Control, WT, and OS58 groups. A detailed description of these types of plots is provided in Figure 2.

For this class of compounds, the yields were below our expectations. Only within the OS58-treated group, a very modest increase in the recovery (1.3-fold) was observed (Figure 5 and Table 1), which, however, did not reach high statistical significance (*p* = 0.082). The reasons for these results are discussed below.

## 3. Discussion and Conclusions

Enzymatic pretreatments have been shown to favorably impact the extraction of valuable compounds from different vegetable sources in a green and sustainable manner [6,7,8,18]. The extraction of lycopene from tomato waste resulted 3-fold higher in enzyme-treated matrices with respect to untreated controls [19], while the phenolic content released from grape waste increased by more than 25%, with respect to the control [20]. In this work, a new *Bacillus subtilis* strain (OS58), with optimized production of at least two enzymes relevant for EAE (cellulases and xylanases), was developed and applied as a pretreatment before cyclic pressurization extraction for the recovery of valuable secondary metabolites from cauliflower. The performance of OS58 was compared to that of a WT *B. subtilis* strain and a control containing the sterile bacterial growth medium.

The recovery of polyphenols, and in particular, of chlorogenic acid and catechins (epigallo catechin gallate, epicatechin, and epicatechin gallate), was enhanced by the pretreatment procedure using the enzymatic mixture derived from the WT strain, and in all cases but one, it further improved with the enzymatic mixture derived from the optimized OS58 strain, demonstrating that the efficiency of the extraction was proportional to the enzymatic activity applied (Table 1). It is worth recalling that no organic solvents were used, avoiding the generation of toxic or polluting waste and preserving the green character of the extraction. Concurrently, the choice of applying a raw culture supernatant of a cellulolytic bacterium such as *B. subtilis*, avoiding expensive commercial enzymes, is a cost-effective strategy that guarantees the economic viability of the process. Moreover, being a soil microorganism, *B. subtilis* grows efficiently over a wide range of conditions and media and can even be fed with agro-industrial waste [21]. *Bacillus subtilis* is ideally suited to industrial applications for the above-mentioned characteristics, and because it secretes many different enzymes directly in the medium, simplifying their recovery [9,10,13,16]; it is also considered a Plant Growth-Promoting Bacterium (PGPB), shown to exert several beneficial effects both on plants and on soil quality [11,22]. For the above reasons, the entire process might be conceived as part of a complex biorefinery which includes: (i) agricultural production of vegetables and their processing before commercialization, (ii) recovery of natural bioactive compounds from vegetable waste, (iii) the use of part of this waste as feedstock for bacterial enzyme production, supporting the compounds’ extraction procedure, (iv-a) application of the post-extraction biomass on agricultural fields to enhance productivity, and (iv-b) alternatively, the post-extraction biomass waste can be used for clean energy production. Indeed, with the introduction of steps iv-a or iv-b, the entire process would be in compliance with the principles of circular economy.

The sustainability of the extraction procedure described here is further enhanced by the fact that the vegetable material used was a waste generated from a real agri-food supply chain because it simply did not meet the aesthetic quality standard requirements for large-scale retail channels. Cauliflowers from this source were collected over three different seasons (spring, summer, and early autumn 2021), and originated from farms located in different geographical areas in Italy. It is known that the content of several secondary metabolites in fruits and vegetables is strongly dependent on several parameters, such as specific cultivar, seasonal harvesting time, cultivation site, endogenous circadian rhythms, and soil and pest-control strategies [23,24,25,26]. Moreover, during the wheeled transport to the processing warehouse, the produce reached different maturation stages, also related to seasonal conditions. The uncontrolled origin of the experimental material granted it an intrinsic heterogeneity that could not be overcome. These variables heavily impact the composition and overall content of the bioactive compounds of vegetables and are partly responsible for the low statistical significance of some of the data.

Moreover, the solubilisation, and thus, the extraction efficiency, of some compounds occurs in polar organic solvents, which were not used in this work. For example, isothiocyanates, relevant anticancer compounds abundantly present in Brassicaceae [27], are usually extracted from fresh material using organic solvents, and even in those cases, with extremely variable returns, as reported in the literature [28,29,30]. In general, however, organic solvents guarantee much higher yields with respect to those reported in this work (Table 1). Only Wang et al. [31] reported a low content of isothiocyanates (0.023 µmol/g) in raw cauliflowers extracted using the cooking water. This value is slightly lower than the result obtained in this work, corresponding to 0.027 µmol/g (considering the Mw of 1,3-Benzodithiole-2-thione as 1843 g/mol). Indeed, in our laboratory, by using methanol, isothiocyanates could be recovered from the frozen material 5-fold more efficiently than through the sustainable procedure herein described, with or without the enzymatic pretreatment (Doria E., unpublished data). Nevertheless, the extraction procedure might require further adjustments for some classes of compounds. For instance, the prolonged defrosting process (48 h) carried out at room temperature (20 °C–23 °C), might compromise the recovery of highly volatile and intrinsically unstable bioactive molecules.

It is important to highlight that, as opposed to most of the literature data, this work was carried out on a pilot scale, using 5 kg of raw material for each sample and for each of the four experimental replicates. To our knowledge there are no previous reports on such large-scale extractions.

In conclusion, the OS58-based sustainable extraction procedure was demonstrated to be more effective than the WT strain-based extraction in the recovery of bioactives from *Brassica oleracea L. conv. botrytis* (L.) *Alef. var. botrytis* L.; the unpurified bacterial enzymes notably improved the retrieval of valuable phytochemicals from the matrix in a concentration-dependent manner. The applicability of the promising sustainable enzymatic pretreatment, coupled with solid-liquid extraction at high pressure, is currently being tested on raw vegetable materials with different structural characteristics and bioactives composition, to validate the applicability of raw enzymatic mixtures for nutraceuticals recovery.

## 4. Materials and Methods

### 4.1. Bacterial Strains 

*Bacillus subtilis* strains used in this study are a WT strain and its derivative, OS58. The WT derives from PB5249, a *swrA*^+^ spontaneous derivative [32] of the auxotrophic *trpC2 pheA1* strain JH642 (GenBank accession no. CM000489.1) [15], which was sequentially transformed with the PCR products of the *trpC* and *pheA* genes, obtained from the genomic DNA of the wild NCIB 3610 strain (GenBank accession no. CP020102.1). Amplifications of the *trpC* and *pheA* genes were performed using the primer pairs trpC_Up 5′-AGTGAAAACACTGGTTCTGCCG-3′ and trpC_Dw 5′-GATGGATTGCTTTACGCTGAGAAG-3′ followed by pheA_Up 5′-AACAGCCTTTGCCAATCGTGGG-3′ and pheA_Dw 5′-GTATACATGGATGCAGCCGCTCAG-3′, respectively. For the first transformation, the selection of the *trpC*^+^ prototroph occurred on minimal medium containing 1.5% agar, 1 mg/mL glucose and phenylalanine (50 µg/mL). Subsequently, *pheA*^+^ transformants were selected on minimal medium without amino acids.

OS58 was obtained from the WT strain by genetic engineering. A patent application is under preparation for this strain; for this reason, the details of the engineering design are undisclosed herein.

### 4.2. Bacterial Growth 

For cellulase and xylanase production, *B. subtilis* WT and OS89 spores were revitalized on LB (Difco Laboratories, New York, NY, USA) 1.5% agar plates and incubated overnight at 37 °C. Isolated colonies were inoculated in Antibiotic Medium 3 (Difco Laboratories) containing 5 mg/mL glucose and grown 16 h at 37 °C with shaking. In the same medium, a second pre-inoculum was set at optical density of 600 nm (OD_600_) 0.2 and grown for 6 h at 37 °C with shaking. The synthetic medium CMD (containing, per liter: 13.67 g Na citrate; 10 g glucose; 7 g NH_4_Cl; 0.5 g MgSO_4_·7H_2_O; 0.5 g K_2_HPO_4_; 0.15 g CaCl_2_·2H_2_O; 0.104 g MnSO_4_·H_2_O; 0.04 g FeCl_2·_6H_2_O; pH6.5) was inoculated at OD_600_ 0.2 and grown for 24 h at 37 °C with 150 rpm orbital shaking. Bacterial growth was followed by OD_600_ readings. Culture supernatant was collected after 40′ centrifugation at room temperature at 2268× *g*. The spent medium (500 mL) was immediately used for the maceration of 5 kg-smashed cauliflowers. 

### 4.3. Cellulase and Xylanase Activity Assays

Cellulase (endo-1,4-β-glucanase; EC 3.2.1.4) and endo-xylanase (endo-1,4-β-xylanase; EC 3.2.1.8) activities were assayed in an aliquot of the culture supernatants after acidification, as previously described [33], using CellG3 and XylX6 Assay Kits (Megazyme^®^), following the protocol provided by the manufacturer.

### 4.4. Plant Material

Fresh cauliflowers (*Brassica oleracea* L*. conv. botrytis* (L.) *Alef. var. botrytis* L.) were obtained, during spring, summer, and autumn 2021, from a food processing plant that collects vegetables from farms located in southern and northern Italy. The fresh material was weighted, aliquoted, and stored at −45 °C ± 1 °C (monitored by a datalogger, Testo 184 T4, Liebherr) for over one month. For each treatment (including controls) and for each of the four experimental replicate, aliquots of 5 kg each (fresh weight before freezing) were thawed at room temperature for 2 days. After draining the residual water, each whole cauliflower, including flowers, leaves and stems, was cut into four parts and ground using an industrial mill (M100, Enoitalia s.r.l., Florence, Italy); the material was completely shattered into small pieces (0.5–1.0 cm in diameter) for subsequent processing. The set of extraction experiments (conducted with the supernatant of the WT strain, the OS58 supernatant, or the sterile medium as a control) was independently carried out four times, on four different lots of cauliflowers.

### 4.5. EAE Pretreatment Procedure of the Cauliflower Waste Material

To 5 kg of fresh material, ground as previously described, 500 mL of bacterial culture supernatant was added, and the final volume was brought to 5 L with water. The suspension was acidified with 85% phosphoric acid to pH 5.0 and was incubated for 16 h at 50 °C with 40 rpm orbital shaking (LOM-7450 Incubator, MRC, London, UK). For each of the four replicates, the material was incubated with 500 mL of sterile CMD instead of the spent medium, as a negative control.

### 4.6. Extraction Process

An isothermal cyclically pressurized extraction process (rapid solid–liquid dynamic extraction) was applied to pretreated cauliflowers using the Naviglio mechanical extractor (Nuova Estrazione, Naples, Italy). The principle is based on cyclic compression and decompression phases that exert a suction effect on extractable compounds present in the vegetable matrix [34]. The solid material was placed into a 50 μm-filtering membrane bag, inserted together with the maceration liquid, into the pressure gradient cylinder that was then filled with deionized water, up to a total volume of 35 L. Each extraction cycle consisted of a 3 min static phase, followed by a 2 min dynamic phase; the number of cycles was 60, for a total time of 5 h. At the end of the extraction, the bag with the solid residue was removed and squeezed, and the aqueous liquid was recovered in a tank. One liter of the collected extract was dried using a rotary evaporator (Rotavapor System R-300, Büchi Labortechnik AG, Flawil, Switzerland); the solid residue was suspended in 50% methanol and filtered using 0.22 µm nylon filter before analyses.

### 4.7. Total Polyphenol Content

The total content of polyphenols was measured using the Total Polyphenols Colorimetric Assay Kit (Steroglass, Perugia, Italy) according to the manufacturer’s instructions. Absorbance was measured at 725 nm and results were expressed in gallic acid equivalents using a gallic acid standard curve.

### 4.8. Catechins and Chlorogenic Acid HPLC Analyses

A 20-µL aliquot of the filtered sample was injected into the HPLC pump (Kontron 420; Kontron Instruments, Munich, Germany) equipped with a C18 column (ZORBAX ODS 250 × 4.6 mm column, 5 µm particle size, Sepachrom, Milan, Italy). For catechins and chlorogenic acid, HPLC analyses were performed with a 0.8 mL/min flow rate and setting the detector at 280 nm; the mobile phases consisted in 5% acetic acid (A) and pure methanol (B), and the chromatographic gradient conditions are summarized in Table 2: 

### 4.9. Isothiocyanates HPLC Analyses

Isothiocyanates were analyzed by HPLC after cyclocondensation, according to the protocol described by Tang et al. [28], with modification. For each sample, an aliquot of 250 µL of extract was mixed with 250 µL of 100 mM potassium phosphate buffer (pH 8.5) and 500 µL of 10 mM 1,2-benzenedithiol in methanol. The reaction mixture was incubated for 2 h at 65 °C and then cooled at room temperature. The mixture was centrifuged at low speed and finally filtered with a 0.22 µm nylon filter before being injected into the HPLC. In this process, 1,2-benzenedithiol reacts with the carbon atom of the –N = C = S group of isothiocyanates to form a five-membered 1,3-benzodithiole-2-thione and the corresponding amine. Using RP-HPLC with an isocratic mobile phase of 80%methanol, a flow rate of 1 mL/min, at 40 °C and UV 365 nm, 1,3-benzodithiole-2-thione can be eluted, providing an analytical measure of the total isothiocyanates present [35]. Different concentrations of allyl-isothiocyanate were used as standards for the calibration curve.

### 4.10. Statistical Analysis

The variation in the amount of the compound extracted among the three experimental conditions, considered as response variable, was explored by using a series of linear mixed models (LMMS). Each model had a common fixed effect, represented by the experimental treatment adopted (factor with three levels: control, WT, OS58), and a common random effect (random intercept) represented by the different samples of organic matter (i.e., replicates). The estimated means, related confidence intervals, and planned comparison among treatments were computed using the emmeans and contrast functions of the emmeans package (version 1.7.2) [17]. All model assumptions were explored by checking residuals distribution against fitted values (Tukey–Anscombe plot) and residual normality against theoretical normal distribution (quantile-quantile plot). Percentual changes with respect to the control were obtained from the raw data. 

All statistical analyses were performed in R (R Core Team, 2021. R: A language and environment for statistical computing. R Foundation for Statistical Computing, Vienna, Austria) [36].

## Figures and Tables

**Table 1:** The concentration of secondary metabolite extracted (mg or μg per gram of fresh cauliflower) for each pretreatment group [control = sterile medium; WT = spent medium from the WT strain; OS58 = spent medium from the optimized strain].

	Control	WT	OS58
Polyphenols (mg/g)	0.176 ± 0.020	0.227 ± 0.007	0.245 ± 0.023 *
Epigallocatechin gallate (g/g)	1.094 ± 0.185	2.170 ± 0.829	3.071 ± 0.740 **
Epicatechin (g/g)	4.685 ± 0.778	8.670 ± 1.346 *	11.30 ± 2.769 **
Epicatechin gallate (g/g)	3.293 ± 0.573	4.411 ± 0.590 **	4.108 ± 0.469 *
Chlorogenic acid (g/g)	0.928 ± 0.658	1.880 ± 0.776 **	2.711 ± 0.871 ***
Isothiocyanates (g/g)	0.004 ± 0.001	0.005 ± 0.001	0.006 ± 0.001

A descriptive analysis of the data—mean values ± standard error of the mean (SEM)—was conducted to describe the difference of the treated groups vs. control. The asterisks (*) indicate the significant differences, obtained by the Linear Mixed Models (*p* < 0.05 *; *p* < 0.01 **; *p* < 0.001 ***).

**Table 2:** The mobile phase gradient.

Time (Min)	A (%)	B (%)
1	90	10
5	90	10
7	80	20
8	80	20
10	75	25
15	70	30
20	20	80
25	50	50
28	70	30
30	90	10

## Data Availability

The data presented in this study are available on request from the corresponding author. The data are not publicly available due to the privacy statement in the original project.
